# A Novel Seedless TSV Process Based on Room Temperature Curing Silver Nanowires ECAs for MEMS Packaging

**DOI:** 10.3390/mi10060351

**Published:** 2019-05-28

**Authors:** Min Meng, Lijuan Cheng, Kai Yang, Mingyan Sun, Yi Luo

**Affiliations:** 1 Microsystem & Terahertz Research Center, China Academy of Engineering Physics (CAEP), Chengdu 610200, China; mengmin@mtrc.ac.cn (M.M.); chenglijuan@mtrc.ac.cn (L.C.); yangkai@mtrc.ac.cn (K.Y.); 2 Institute of Electronic Engineering, China Academy of Engineering Physics (CAEP), Mianyang 621900, China; 3 Institute of Machinery Manufacturing Technology, China Academy of Engineering Physics (CAEP), Mianyang 621900, China; yanmingsun18@163.com

**Keywords:** seedless TSVs process, room temperature curing, electrically conductive adhesives, MEMS packaging, flexible microsystems

## Abstract

The through-silicon-vias (TSVs) process is a vital technology in microelectromechanical systems (MEMS) packaging. The current via filling technique based on copper electroplating has many shortcomings, such as involving multi-step processes, requiring sophisticated equipment, low through-put and probably damaging the MEMS devices susceptible to mechanical polishing. Herein, a room temperature treatable, high-efficient and low-cost seedless TSV process was developed with a one-step filling process by using novel electrically conductive adhesives (ECAs) filled with silver nanowires. The as-prepared ECAs could be fully cured at room temperature and exhibited excellent conductivity due to combining the benefits of both polymethyl methacrylate (PMMA) and silver nanowires. Complete filling of TSVs with the as-prepared 30 wt% silver nanowires ECAs was realized, and the resistivity of a fully filled TSV was as low as 10^−3^ Ω·cm. Furthermore, the application of such novel TSV filling process could also be extended to a wide range of different substrates, showing great potential in MEMS packaging, flexible microsystems and many other applications.

## 1. Introduction

As MEMS packaging evolves into miniaturization, TSV (through-silicon-vias) becomes a critical interconnection technology for MEMS packaging [1,2,3,4,5,6,7,8,9]. The current via-filling technique based on copper electroplating normally involves multi-step processes, including physical vapor deposition (PVD) or chemical vapor deposition (CVD) of barriers and copper seed layers, electroplating of copper plugs and the chemical mechanical polishing (CMP) process to remove the overburden layer and flatten the surface. This via-filling technique has been widely applied in TSV fabrication process benefiting from its low resistivity, capability of void-free fill, high reliability, and compatibility with the thermal budget [10,11,12,13,14,15,16,17,18,19]. However, the technique suffers from many limitations of either relying on sophisticated equipment (PVD, CVD, CMP, etc.), an unrealistic amount of pinch-off time, and sensitivity to process conditions under complex process parameters, as well as providing the risk of damage to the sensitive MEMS device if the via is fabricated after the device is finished. All these defects mentioned above make copper electroplating an unsuitable tool for TSV fabrication. Thus, it is essential and vital to explore a seedless, high-efficient and low-cost TSV fabrication method for MEMS packaging.

In recent years, great efforts have been made to improve the TSV fabrication process. The focus is mainly on the research of new filling methods and materials, including seedless electroplating [20,21], liquid-metal injection (SnZn, SnAu) [22,23], metal paste printing (Ag, Au, Cu) [24,25,26,27,28], etc. Among these improved methods, interest in liquid-metal injection and paste printing is currently growing apace and great progress has been made. For instance, Jiebin Gu et al. developed an efficient TSV technology based on liquid-metal injection with a customized nozzle wafer for MEMS packaging [23]. Lee Mi Do and co-workers reported a cost-effective via metallization method using a nano-Ag particle solution through four cycles of printing and heat evaporation [24]. Nevertheless, the aforementioned TSV fabrication methods are limited by complex processing steps, drastically reducing the efficiency of fabrication and universality of the approaches. Moreover, the heat curing process or high temperature treatment is also indispensable, which inevitably brings negative effects on performance of the devices susceptible to high temperature and limits their application in flexible microsystems. However, the question of how to realize vias in a flexible substrate is still a critical research topic, owing to the growing demand in internet of things (IOT) technologies for various applications [26,29,30,31], such as smart grid, food safety monitor, body sensor network, etc.

Silver nanowires, as one of the most important conductive materials, have attracted extensive attention for many applications, one of which is as a conductive filler in ECAs [32,33,34,35,36]. It is reported that silver nanowires are able to provide ECAs with similar conductivity to traditional Ag flakes at much lower filler loading, meaning they can more easily form a continuous network. In this paper, we developed a room temperature treatable, high-efficient and seedless TSV process for MEMS packaging by using a kind of novel ECAs filled with 30 wt% silver nanowires. The resistivity of a fully filled TSV was as low as 10^−3^ Ω·cm by orders of magnitude, which was applicable for MEMS packaging electrically. Additionally, the TSV process carried out at room temperature with a one-step filling process in a short time also shows universal in via metallization and great potential in flexible microsystems.

## 2. Materials and Methods 

### 2.1. Chemicals and Materials

Silver nitrate (AgNO_3_, 99%), sodium chloride (NaCl, 99%), poly (vinylpyrrolidone) (PVP, K30, Mw ≈ 40,000), propanetriol (C_3_H_8_O_3_, 99%) were purchased from Sinopharm Chemical Reagent Co., Ltd. (Shanghai, China). Ethanol (C_2_H_5_OH, 99.9%), acetone (C_3_H_6_O, 99.9%), ammonium hydroxide (NH_4_OH, 99.9%), hydrogen peroxide (H_2_O_2_, 99.9%), hydrofluoric acid (HF, 99.9%) and ammonium fluoride (NH_4_F, 99.9%) were purchased from Chengdu Cologne Chemical Co., Ltd. (Chengdu, China). Polymethyl methacrylate (PMMA, average M_w_: 950,000; T_g_: 105 °C) were purchased from ALLRESIST (Strausberg, Germany). All chemical reagents were used as received without further purification. Ultrapure water (18.2 MΩ·cm) was used as the solvent and cooling water.

### 2.2. Synthesis of Silver Nanowires

The silver nanowires were synthesized through a modified polyol process reported previously [37]. Specifically, 120.0 mL of propanetriol, 150.0 mL of a propanetriol solution containing PVP (60 mg/mL), 15.0 mL of a propanetriol solution containing NaCl (100 mM) and 14.1 mL of a propanetriol solution containing AgNO_3_ (1 M) were added in a 1000-mL three-necked flask at room temperature. After the flask had been capped with glass stoppers, the solution was magnetically stirred at room temperature for about 5 min. Subsequently, the capped flask was transferred into a heating jacket and heated to 210 °C under magnetic stirring. Afterwards, the solution was slowly added into equivalent cooling water. The mixed solution was stilled at room temperature overnight. Silver nanowires were purified to remove excess precursors, PVP, and NaCl by conducting filtration three times in the presence of water and ethanol. The silver nanowires were finally dispersed into a mixture of ethanol and acetone for future use. The concentration of silver element measured by Inductively Coupled Plasma-Atomic Emission Spectrometry (ICP-AES, Atomscan Advantage, Thermo Jarrell Ash Co., Boston, MA, USA) was 5.1 mg/mL.

### 2.3. Preparation and Conductivity Measurement of Silver Nanowires ECAs

In a typical procedure, a certain amount of the purified silver nanowires was incorporated into the PMMA matrix with stirring for about 2 h to make the fillers uniformly dispersed in matrix and meanwhile decrease the amount of solvents. To determine the appropriate amount of silver nanowires, the ECAs filled silver nanowires with 20, 30, and 40 wt% loadings were prepared, respectively. Two strips of polyimide tape were applied onto a pre-cleaned glass slide with a gap width of 1 cm. The formulated ECAs was bladed into the space between the two strips. The polyimide tapes were removed before curing. The resistance of silver nanowires ECAs with different loadings was monitored every 15 min by multimeter (LINI-T UT58D, Uni-Trend Technology Co., Dongguan, China) in a 2-wire configuration until the resistance began to hardly change. The resistivity *ρ* of the fully cured silver nanowires ECAs was calculated by the Equation (1):

(1) $$\rho =R_{s}*\omega ,$$

where *R_s_* and *ω* are the sheet resistance and thickness of a sample, respectively. The sheet resistance *R_s_* was measured by Non-Contact Resistivity Measurement System (Leihighton 1510EB, Semilab LEI, Pennsylvania, MA, USA), and the average thickness ω of a sample film was obtained with a stylus profiler (KLA-Tencor P-7, KLA-Tencor Co., Milpitas, CA, USA).

### 2.4. Fabrication of the Novel TSVs Process

**Figure 1:**
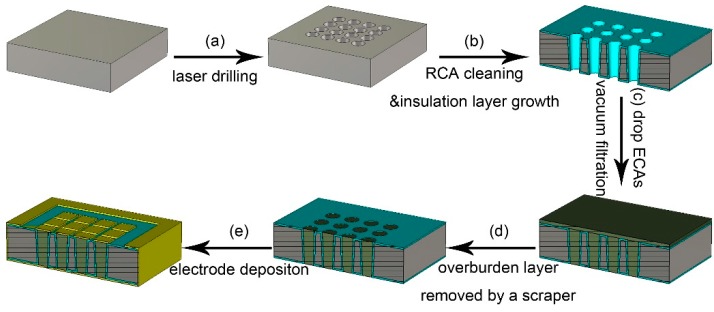
Schematic images of the TSVs fabrication process.

The novel seedless TSV process based on the as-prepared room temperature curing ECAs is illustrated in Figure 1. The TSVs with an inlet diameter of 100 μm and a depth of 625 μm on a six-inch silicon wafer were attained by laser drilling (DPD02, Suzhou Delphi Laser Co., Ltd., Suzhou, China, Figure 1a). The patterned wafer was cleaned with standard RCA process (first, cleaned with 1:1:5 NH_4_OH:H_2_O_2_:H_2_O for 15 min at 75 ± 5 °C, quick dump rinse; second, cleaned with 1:10 HF:NH_4_F for 2 min at room temperature, quick dump rinse; finally, dried with nitrogen). Subsequently, the 320 nm SiO_2_ insulation layer was grown thermally by high temperature oxidation diffusion furnace (M5112-5/UM, The 48th research institute of China electronics technology group Co., Changsha, China, Figure 1b). Then the patterned wafer was diced into 2 × 2 cm^2^ specimens with 4 × 4 TSVs array for future use. Afterwards, a certain amount of the as-prepared sliver nanowires ECAs was dropped onto a specimen and then the sample was put into a vacuum filtration system (Figure 1c). Subsequently, the vacuum pump was turned on to perform the filling process. After the process was finished, the sample was taken out and was cured at room temperature for a certain time. After being fully cured, the overburden layer was easily removed by a scraper (Figure 1d). A small amount of residue on the surface of the filled TSV substrate was gently wiped off with clean cloth dipped in a small amount of anhydrous ethanol and then the substrate was dried with nitrogen. The surface of the substrate was characterized by a super depth of focus microscope (VHX-5000, KEYENCE, Osaka, Japan). Pad metals with 20 nm Ti/80 nm Au were finally deposited on the front and back of the substrate by sputtering. The front pads were deposited with a shadow mask that was obtained by laser drilling with 2 × 2 mm^2^ square patterns (Figure 1e). The resistance of filled TSVs was measured by a manual analytical probe station equipped with a four-point probe. The profiles of the filled TSVs cured at room temperature for a certain time were examined by scanning electron microscope (SEM, FEI Co., Hillsboro, OR, USA).

### 2.5. Characterizations

SEM images were collected on a FEI Nova NanoSEM 650 field emission scanning electron microscope (FEI Co., Hillsboro, OR, USA) operating at 15 kV accelerating voltage. Optical images were collected on a VHX-5000 super depth of focus microscope (KEYENCE, Osaka, Japan). ICP-AES (Atomscan Advantage, Thermo Jarrell Ash, Waltham, MA, USA) was used to determine the concentration of silver. X-Ray Diffraction (XRD) characterization was performed using a X’Pert Pro X-ray diffractometer (PANalytical B.V., Almelo, The Netherlands) with a monochromatized Cu Kα radiation source and a wavelength of 0.1542 nm. The sheet resistance of silver nanowires ECAs coated on a glass slide was measured on a Leihighton 1510EB Non-Contact Resistivity Measurement System (Semilab LEI, Lehighton, PA, USA). The average thickness of a sample film was obtained with a KLA-Tencor P-7 stylus profiler (KLA-Tencor Co., Milpitas, CA, USA). A KEYSIGHT B29021 sourcemeter (KEYSIGHT TECHNOLOGIES Co., Santa Rosa, CA, USA) attached to a LAB150 manual analytical probe station (Shenzhen zhanxin technology Co., Ltd., Shenzhen, China) was used for electrical characterization of fully filled TSVs.

## 3. Results and Discussion

### 3.1. Characterization of Silver Nanowires

**Figure 2:**
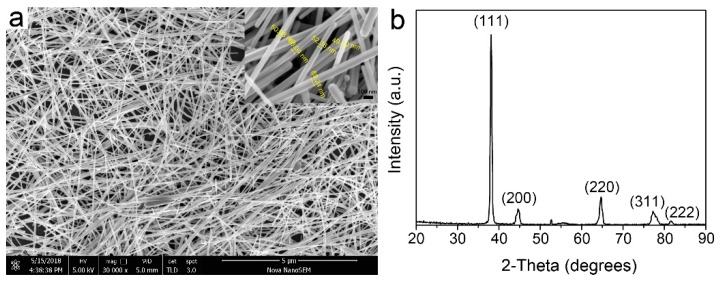
(**a**) SEM images of silver nanowires after being purified and (**b**) XRD spectrum of the synthesized silver nanowires.

Figure 2a shows a typical SEM image of the synthesized silver nanowires. The inset is the local amplification image. The SEM images show that the as-prepared silver nanowires have a length of about 6~13 μm and diameter of 50~90 nm. Rare Ag nanoparticles were observed in Figure 2a (purity > 95%). Moreover, through purifying silver nanowires using the vacuum filtration method, the breakage of silver nanowires could be dramatically avoided. The face-centered cubic (fcc) structure of the silver nanowires was revealed by the XRD spectrum in Figure 2b. Five diffraction peaks could be observed and indexed to the (111), (200), (220), (311) and (222) planes, respectively. The lattice constant calculated from these XRD patterns was 4.0812 Å, which was close to the reported data 4.0862 Å (Joint Committee Powder Diffraction Standards, JCPDS file 04-0783).

### 3.2. Morphology and Electrical Properties Analysis on the Novel ECAs

**Figure 3:**
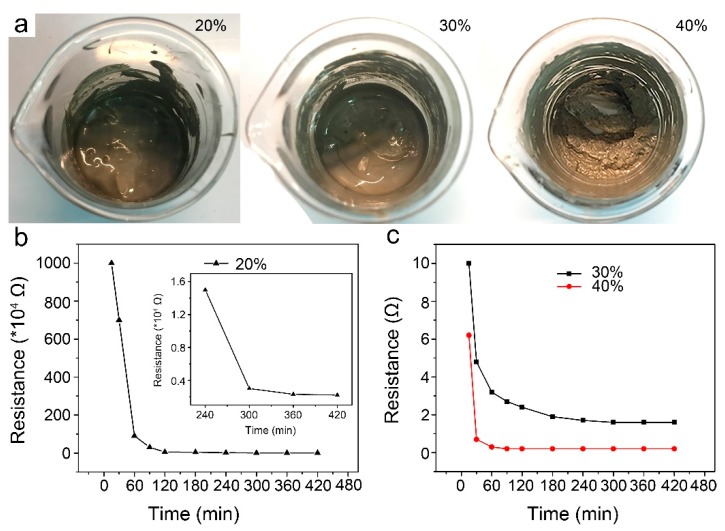
(**a**) Photos of the ECAs filled silver nanowires with 20, 30, and 40 wt% loadings. (**b**,**c**) The variations in resistance of ECAs filled silver nanowires with different loadings during curing at room temperature for 7 h: (**b**) 20 wt%, (**c**) 30 and 40 wt%. The inset in Figure 3b shows the locally magnified curve.

The photos of the ECAs filled silver nanowires with different loadings are represented in Figure 3a. When the loading was 20 wt%, silver nanowires could be uniformly dispersed in the PMMA matrix. As the loading of silver nanowires was increased to 30 wt%, the dispersibility of silver nanowires in the PMMA matrix was kept very well. While the loading continuing to be increased to 40 wt%, silver nanowires were difficult to be dispersed in the PMMA matrix, and the ECAs became less viscous and flowing.

The resistance of ECAs is one of the most important parameters relating to interconnect reliability. Hence, in-situ monitoring of the variation in resistance of the as-prepared ECAs was undertaken at room temperature, as shown in Figure 3b,c. For ECAs filled with 20 and 30 wt% silver nanowires, the resistance was so high before curing that it was beyond the range of the multimeter. However, for the ECAs filled with 40 wt% silver nanowires, the resistance was monitored to be about 260 Ω before curing, indicating that the higher content of silver nanowires led to easier formation of the conductive networks. When curing was proceeded to 15 min, the resistance of the ECAs filled with 20 and 30 wt% silver nanowires was decreased to 10 MΩ and 10 Ω, respectively. The resistance of the ECAs filled with 20 wt% silver nanowires continued decreasing to 3 kΩ at 5 h and had little change from 5 to 7 h, signifying that the effective conductive networks had been established when curing proceeded to 5 h. According to Figure 3c, we could similarly deduce that for ECAs filled with 30 and 40 wt% silver nanowires, the effective conductive networks were established when curing proceeded to 3 and 1 h, and the corresponding resistance was 1.7 and 0.2 Ω, respectively. As such, it can be presumed that the higher silver nanowires contents are, the more conductive paths are formed in a shorter curing time, which is consistent with the literature [33,34].

**Figure 4:**
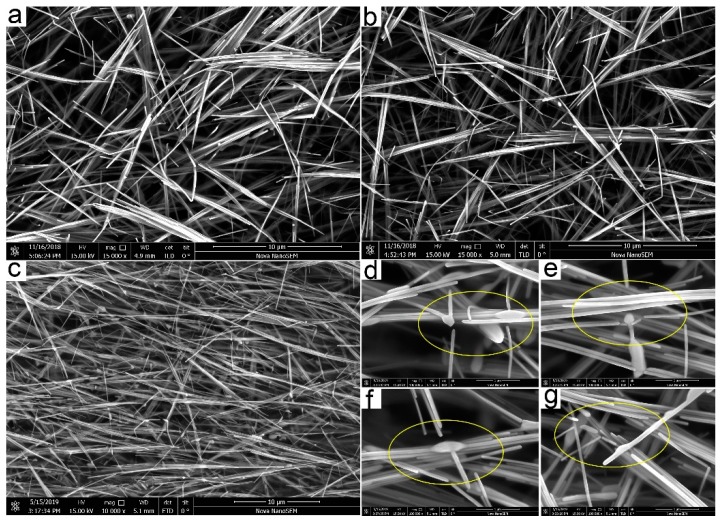
SEM images of the ECAs filled with 30 wt% silver nanowires before (**a**) and after (**b**) being heated at 180 °C for 20 min in a vacuum oven. (**c**) SEM image of the ECAs cured at 250 °C for 20min. (**d**–**g**) Localized magnified SEM images of sintering and fracture in the silver nanowires shown in (**c**).

To verify whether the complete curing had been carried out at room temperature and the compatibility of the room temperature cured ECAs with a subsequent low- or high-temperature bonding process, the cured ECAs filled with 30 wt% silver nanowires continued to be cured at 180 °C and 250 °C for 20 min in a vacuum oven, respectively. The resistance was measured to be 1.1 Ω after 180 °C heat curing, showing little change. The SEM images in Figure 4a,b show the conductive networks before and after the ECAs being cured at 180 °C. There was nearly no difference in the distribution and the morphology of silver nanowires, which further demonstrated that effective conductive networks had been established at room temperature. Based on previous reports [38,39,40], we deduced that it was PMMA which contributed to the cold curing of the as-prepared ECAs and that the silver nanowires accelerated the curing process. Nevertheless, the resistance increased obviously from 1.7 to 20 Ω after 250 °C heat curing. As represented in Figure 4c–g, large amounts of sintering and fracture are observed in the silver nanowires, which leads to localized disruption of the three-dimensional conductive network and an irreversible increase in resistance of the ECAs. As such, we speculated that the room-temperature cured ECAs cannot provide a robust contact with low resistance during the high temperature bonding process (> 200 °C). However, the applications of the ECAs in TSV technology will not be limited due to the low-temperature (< 200 °C) bonding technology innovations that can overcome issues related to the high temperature bonding process, for instance, cracks of thinned and fragile wafer during bonding, performance degradation under higher bonding temperature, serious wafer/chip warpage, bonding misalignment, and compatibility with the back-end-of-line process conditions and materials [41,42,43].

After confirming that the as-prepared ECAs were fully cured at room temperature, we calculated the resistivity to evaluate the conductivity by the Equation (1) depicted in Section 2.3. The resistivity of the ECAs filled with 30 and 40 wt% silver nanowires was 4.8 × 10^−3^ and 6.0 × 10^−4^ Ω·cm, respectively, which was comparable to that of other reported high-quality ECAs [33,34]. Note that, unlike the previously reported ECAs composed of epoxy resin, curing agent, curing catalyst, etc., the ECAs prepared herein combined the benefits of both PMMA and silver nanowires. Such a combination is novel and the ECAs can be fully cured at room temperature and also exhibit excellent conductivity, showing great potential in MEMS packaging and flexible microsystems.

### 3.3. The Seedless TSV Process Based on the As-Prepared ECAs and Properties Analysis on the Fully Filled TSVs

**Figure 5:**
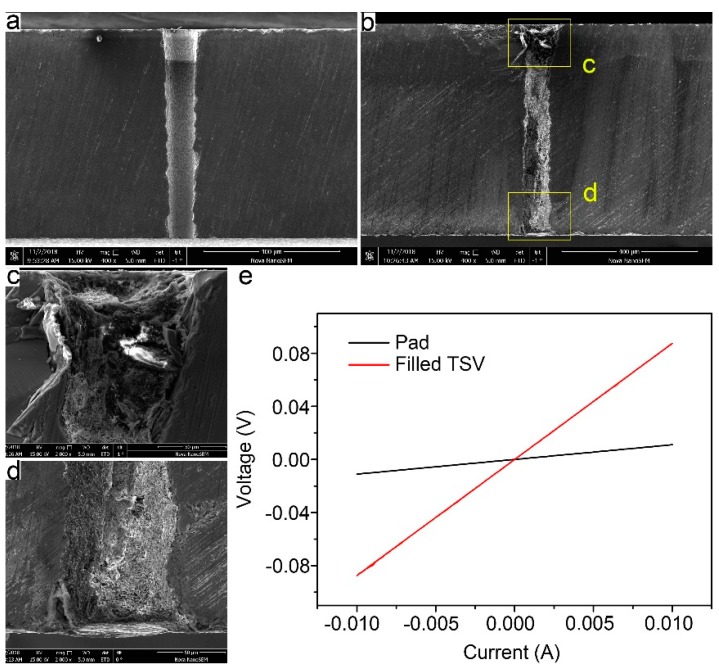
(**a**,**b**) Cross-sectional SEM images of a TSV before (**a**) and after (**b**) being filled with 30 wt% silver nanowires ECAs. (**c**) and (**d**) show the magnified images of top and bottom of the filled TSV marked with boxes in (**b**), respectively. (**e**) I-V measurement curves of a fully filled TSV with 30 wt% silver nanowires ECAs (red) and deposited Ti/Au pad (black).

The excellent fluidity and conductivity of the as-prepared ECAs make them promising candidates for the TSV metallization process. In this work, the complete filling of TSVs with 30 wt% silver nanowires ECAs was realized through a simple one-step vacuum filtration process. Figure 5a shows the cross-sectional SEM images of the fabricated TSV with a SiO_2_ insulation layer, demonstrating a nearly vertical TSV with a depth of about 625 μm and an inlet diameter of about 100 μm. In contrast to TSV applications in other three-dimensional (3-D) integrations, such as memories or ICs, the feature size of TSV in MEMS packaging is relatively large, at up to hundred microns. Therefore, the size we designed here is suitable for MEMS packaging. The profile of a TSV filled with the 30 wt% silver nanowires ECAs after being cured for 3 h at room temperature was confirmed and represented in Figure 5b, indicating the TSV has been fully filled through the facile filtration process. The removal of the overburden layer was simply carried out by a scraper and clean cloth without surface contamination due to the integrality and easy cleaning of fully cured overburden ECAs layer, as shown in Figure S1. Figure 5c,d represents the corresponding magnified images of the regions marked in Figure 5b. Some cracks and voids were observed in the filled TSV, which might adversely affect the electrical conductivity of the filled TSV. Therefore, further investigations are still required to improve viscosity and liquidity of the silver nanowires ECAs, and to perfect the TSVs filling approach. In spite of this, the simple and robust filling method of TSVs with room temperature curing 30 wt% silver nanowires ECAs shows great potential in via metallization.

Figure 5e shows the I-V curves of a fully filled TSV (red) and deposited Ti/Au pad (black), which were obtained by a sourcemeter attached to a manual analytical probe station equipped with a four-point probe. According to the I-V curves, the resistance of a fully filled TSV was calculated to be 7.5 Ω (the resistance of Ti/Au pad had been deducted). That was about twice the theoretical value, which was calculated to be 3.7 Ω by the Equation (2):

(2) R=ρL/S,

where *ρ* is the resistivity of the ECAs filled with 30 wt% silver nanowires, *L* is the depth of the TSV, and *S* is the cross sectional area of the TSV. Nevertheless, the resistance was still applicable for MEMS packaging electrically and the TSV fabrication process shows great potential in the MEMS packaging field.

### 3.4. Extending the Novel Seedless TSV Process to Other Substrates

**Figure 6:**
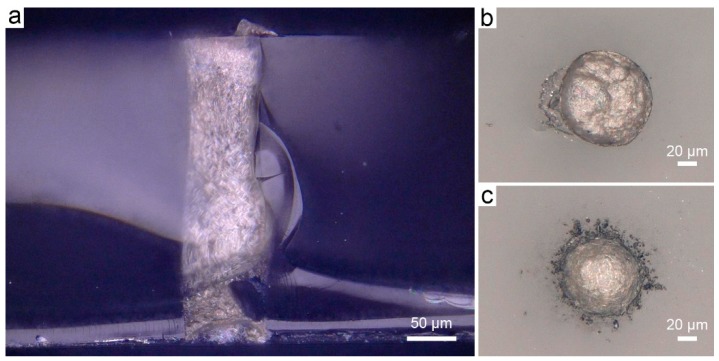
(**a**) The optical magnified cross-sectional image of a TGV filled with the 30 wt% silver nanowires ECAs. (**b**) and (**c**) show that the top and bottom of a fully filled TGV are flat and leveled with the substrate surface.

To demonstrate the universality of the room temperature treatable, low-cost, and high through-put TSV process mentioned above, this process was applied to the vias of other materials, e.g., the through glass vias (TGVs), another attractive technique in MEMS and other 3-D packaging fields [44,45]. Figure 6 represents the profile of a TGV filled with the 30 wt% silver nanowires ECAs and cured for 3 h at room temperature. The via with a depth of about 300 μm and an inlet diameter of about 100 μm was fully filled, as depicted in Figure 6a. In addition, the top and bottom of a filled-via were flat and leveled with the substrate surface, as shown in Figure 6b,c. Then the I-V curve of a fully filled TGV was obtained by using the same test method as that of TSVs, as shown in Figure S2. According to the I-V curves, the resistance of a fully filled TGV was calculated to be 4.3 Ω (the resistance of Ti/Au pad had been deducted), showing a similar resistivity to that of a fully filled TSV. As such, the simple, cost-effective, high through-put and robust filling method of through unknown vias (TXVs) with room temperature curing ECAs shows great potential and universal in via metallization for MEMS and other packaging applications.

## 4. Conclusions

In summary, a typically seedless, high through-put and cost-effective TSV process based on a kind of room temperature curing ECAs for MEMS packaging and flexible microsystems was proposed in this work. The resistivity of the ECAs filled with 30 and 40 wt% silver nanowires was 4.8 × 10^−3^ and 6.0 × 10^−4^ Ω·cm after fully curing at room temperature, respectively, which was comparable to that of other reported high-quality ECAs and is also suitable for MEMS packaging electrically. By carefully controlling the pressure differential between the top and bottom ends of a TSV, the full-filling of TSVs with 30 wt% silver nanowires ECAs was realized through a one-step vacuum filtration process. In particular, this high-efficient, seedless and robust approach could serve as a general strategy for the TXVs process, showing great potential in MEMS and other 3-D packaging fields. Furthermore, the appealing room temperature curing characteristic of the strategy developed here also makes the TXVs approach for prospective candidates in flexible and other microsystems susceptible to high temperature.

## Supplementary Materials

The following are available online at https://www.mdpi.com/2072-666X/10/6/351/s1, Figure S1. (a) Low magnification optical image of the filled TSVs array with overburden layer being removed by a scraper. (b,c) Magnified optical images of the top side of a fully filled TSV randomly selected from (a). (d,e) Magnified optical images of the back side of a fully filled TSV randomly selected from (a), Figure S2. I-V measurement curves of a fully filled TGV with 30 wt% silver nanowires ECAs (red) and deposited Ti/Au pad (black).
