# Supplementary material for: A Novel Seedless TSV Process Based on Room Temperature Curing Silver Nanowires ECAs for MEMS Packaging
Source: Micromachines (Basel). 2019 May 28;10(6):351. doi: 10.3390/mi10060351 (PMC6631795; doi:10.3390/mi10060351)
Supplement: Supplementary file 1 [file micromachines-10-00351-s001.pdf]

micromachines

Article

A Novel Seedless TSV Process based on Room Temperature Curing Silver Nanowires ECAs for MEMS Packaging

Min Meng [1,2], Lijuan Cheng [1,2], Kai Yang [1,2], Mingyan Sun [3], and Yi Luo [1,*]

[1] Microsystem & Terahertz Research Center, China Academy of Engineering Physics (CAEP), Chengdu 610200, China; mengmin@mtrc.ac.cn (M.M.); chenglijuan@mtrc.ac.cn (L.C.); yangkai@mtrc.ac.cn (K.Y.)
[2] Institute of Electronic Engineering, China Academy of Engineering Physics (CAEP), Mianyang 621900, China;
[3] Institute of Machinery Manufacturing Technology, CAEP, Mianyang 621900, China; yanmingsun18@163.com
* Correspondence: luoyi@mtrc.ac.cn; Tel.: +86-028-6572-6021

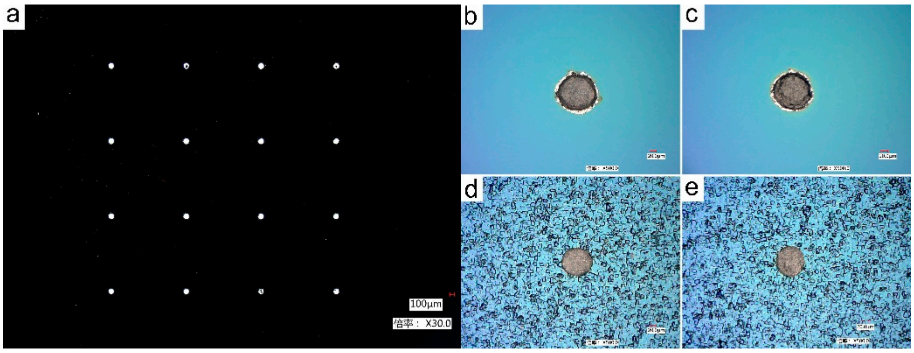

Figure S1. (**a**) Low magnification optical image of the filled TSVs array with overburden layer being removed by a scraper. (**b**,**c**) Magnified optical images of the top side of a fully filled TSV randomly selected from (**a**). (**d**,**e**) Magnified optical images of the back side of a fully filled TSV randomly selected from (**a**).

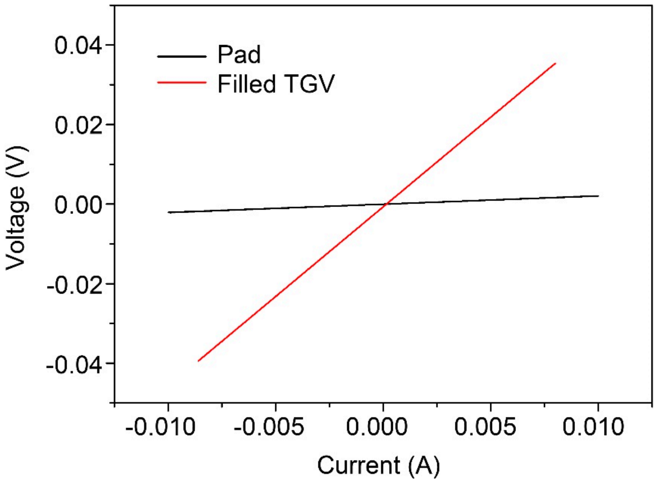

Figure S2. I-V measurement curves of a fully filled TGV with 30 wt% silver nanowires ECAs (red) and deposited Ti/Au pad (black).
